# 4-({(*Z*)-5-[(*Z*)-3-Eth­oxy-4-hy­droxy­benzyl­idene]-3-methyl-4-oxo-1,3-thia­zolidin-2-yl­idene}amino)­benzoic acid dimethyl­formamide monosolvate

**DOI:** 10.1107/S1600536812047307

**Published:** 2012-11-24

**Authors:** Paul Kosma, Edgar Selzer, Kurt Mereiter

**Affiliations:** a Department of Chemistry, University of Natural Resources and Life Sciences, Muthgasse 18, A-1190 Vienna, Austria; b University Clinic of Radiotherapy, Medical University Vienna, Währinger Gürtel 18-20, A-1090 Vienna, Austria; c Institute of Chemical Technologies and Analytics, Vienna University of Technology, Getreidemarkt 9/164SC, A-1060 Vienna, Austria

## Abstract

The mol­ecular structure of the title compound, C_20_H_18_N_2_O_5_S·C_3_H_7_NO, represents an essentially planar 5-benzyl­idene-thia­zolidine moiety (r.m.s. deviation from planarity without ring substituents = 0.095 Å) to which the 4-amino­benzoic acid fragment is inclined at 76.23 (1)°. In the crystal, the benzoic acid mol­ecules are arranged in layers parallel to [001] which are built up from inversion dimers held together by head-to-tail phenol–carb­oxy O—H⋯O hydrogen bonds and head-to-tail π–π stacking inter­actions between the 5-benzyl­idene-thia­zolidine moieties (ring centroid distance = 3.579 Å). These layers are separated by the dimethyl­formamide solvent mol­ecules which are firmly anchored *via* a short O—H⋯O hydrogen bond [O⋯O = 2.5529 (10) Å] donated by the –COOH group.

## Related literature

For bioactive compounds based on the 4-thia­zolidinone scaffold of the title compound, see: Ottanà *et al.* (2005 ▶); Verma & Saraf (2008 ▶). For potential anti­cancer activity *via* α_v_β_3_ integrin antagonistic properties of 4-thia­zolidinone derivatives, see: Dayam *et al.* (2006 ▶). For a description of the Cambridge Structural Database, see: Allen (2002 ▶). For standard bond lengths, see: Allen *et al.* (1987 ▶). For crystal structures related to that of the title compound, see: Ottanà *et al.* (2005 ▶); Yavari *et al.* (2008 ▶); Deepthi *et al.* (2001 ▶); Tomaščiková *et al.* (2008 ▶).
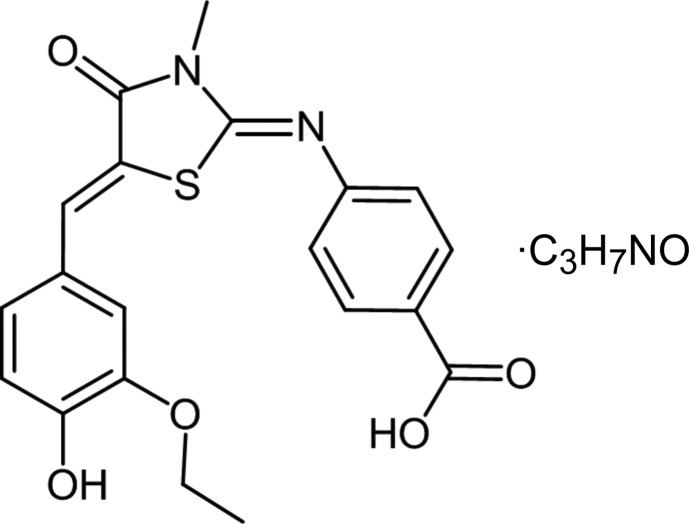


## Experimental

### 

#### Crystal data

C_20_H_18_N_2_O_5_S·C_3_H_7_NO
*M* *_r_* = 471.52
Triclinic, 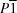

*a* = 7.7532 (3) Å
*b* = 9.3081 (3) Å
*c* = 15.6969 (3) Å
α = 86.390 (2)°
β = 89.813 (2)°
γ = 87.656 (2)°
*V* = 1129.61 (6) Å^3^
*Z* = 2
Mo *K*α radiation
μ = 0.19 mm^−1^
*T* = 100 K
0.53 × 0.35 × 0.24 mm

#### Data collection

Bruker Kappa APEXII CCD diffractometer
Absorption correction: multi-scan (*SADABS*; Bruker, 2008 ▶) *T*_min_ = 0.89, *T*_max_ = 0.96
29040 measured reflections
6537 independent reflections
5993 reflections with *I* > 2σ(*I*)
*R*_int_ = 0.024

#### Refinement

*R*[*F* ^2^ > 2σ(*F* ^2^)] = 0.033
*wR*(*F* ^2^) = 0.094
*S* = 1.02
6537 reflections
304 parameters
H-atom parameters constrained
Δρ_max_ = 0.51 e Å^−3^
Δρ_min_ = −0.22 e Å^−3^

### 

Data collection: *APEX2* (Bruker, 2008 ▶); cell refinement: *SAINT* (Bruker, 2008 ▶); data reduction: *SAINT*; program(s) used to solve structure: *SHELXS97* (Sheldrick, 2008 ▶); program(s) used to refine structure: *SHELXL97* (Sheldrick, 2008 ▶); molecular graphics: *Mercury* (Macrae *et al.*, 2006 ▶); software used to prepare material for publication: *PLATON* (Spek, 2009 ▶) and *publCIF* (Westrip, 2010 ▶).

## Supplementary Material

Crystal structure: contains datablock(s) I, global. DOI: 10.1107/S1600536812047307/fk2066sup1.cif

Structure factors: contains datablock(s) I. DOI: 10.1107/S1600536812047307/fk2066Isup2.hkl

Supplementary material file. DOI: 10.1107/S1600536812047307/fk2066Isup3.cml

Additional supplementary materials:  crystallographic information; 3D view; checkCIF report

## Figures and Tables

**Table 1:** Hydrogen-bond geometry (Å, °)

*D*—H⋯*A*	*D*—H	H⋯*A*	*D*⋯*A*	*D*—H⋯*A*
O5—H5*o*⋯O6	0.84	1.73	2.5529 (10)	167
O3—H3*o*⋯O4^i^	0.84	2.05	2.7386 (11)	139

Symmetry code: (i) 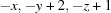.

**Fig. 1.**
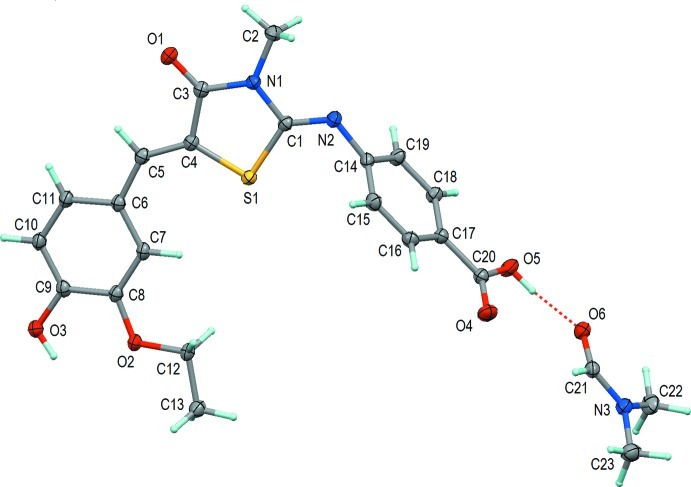
Molecular structure of the title compopund with displacement ellipsoids drawn at the 50% probability level. Dashed red line indicates the -COOH···DMF interaction.

**Fig. 2.**
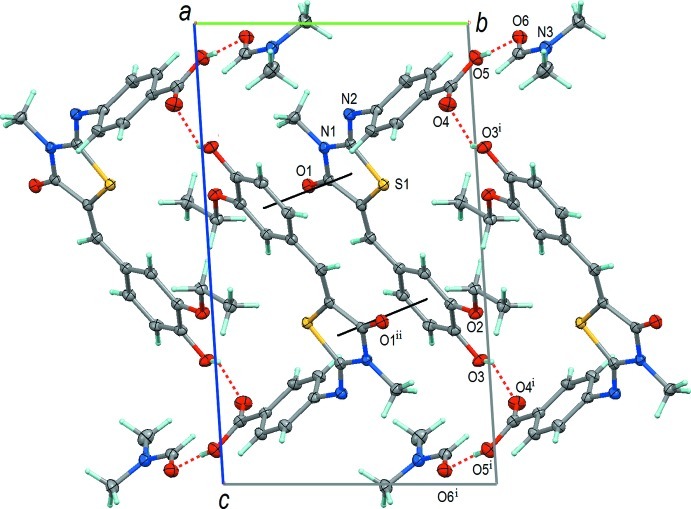
Crystal packing viewed along [100] showing O—H···O hydrogen bonds as dashed red lines and π-π-stacking interactions as black lines. Symmetry codes: (i) -x, 2 - y, 1 - z; (ii) 1 - x,1 - y,1 - z.

**Fig. 3.**
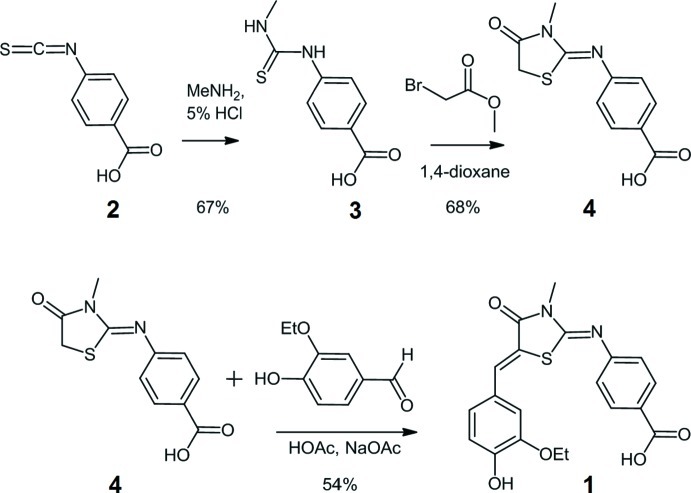
Reaction scheme for the synthesis of 1.

## supplementary crystallographic information

### Comment

Compounds based on the 4-thiazolidinone scaffold received attention in recent years as bioactive agents with a broad therapeutic potential (Ottanà *et al.*, 2005; Verma & Saraf, 2008). The title compound 1.DMF, (I), is the dimethylformamide monosolvate of a 4-thiazolidinone derivative 1, which shows significant anticancer activity *via*α_v_β_3_ integrin antagonistic properties (Dayam *et al.*, 2006). The molecule of 1 consists of a planar thiazolidine ring that is substituted in 2,3,4, and 5-positions by a 4-aminobenzoic acid fragment, a methyl group, an oxo atom O1, and a 3-ethoxy-4-hydroxy-phenylmethylidene group (Fig. 1). Bond lengths and bond angles in the thiazolidine ring correspond well with related compounds, particularly with Cambridge Structural Database (Allen, 2002) refcode CAPGIK (Ottanà *et al.*, 2005; for other structures, see: CSD BIVWUZ (Yavari *et al.*, 2008), OGIBAH (Deepthi *et al.*, 2001), and SIXFOV (Tomaščiková *et al.*, 2008)), where the S1—C1 bond was typically found to be slightly longer than the S1—C4 bond (1.7793 (9) Å and 1.7564 (10) Å in 1). The ring bonds N1—C1 = 1.3873 (12) Å and N1—C3 = 1.3729 (12) Å in 1 are shorter than standard C—N single bonds and indicate some quasi-aromatic conjugation, while C3—C4 1.4824 (13) Å is longer than a typical aromatic C—C ring bond (Allen *et al.*, 1987). The thiazolidine ring and its four substituent atoms N2, C2, O1, and C5 are nearly planar. Their r.m.s. deviation from planarity is 0.021 Å, while for the thiazolidine ring atoms this quantity is 0.007 Å. The phenylmethylidene unit is only moderately inclined to the thiazolidine ring (ring-ring inclination angle 10.85 (6)°) enabling conjugation between the two rings *via* the methylidene carbon C5. The bond angle C4—C5—C6 = 133.55 (9)° is large in response to the short intramolecular contact distance S1···H7 = 2.752 Å. The 4-aminobenzoic acid fragment is inclined to the thiazolidine ring by 75.01 (3)° and the bond angle at the linker atom N2 is C1—N2—C14 = 121.40 (8)°. CSD refcode CAPGIK (Ottanà *et al.*, 2005) shows comparable trends in bond angles and conformation.

In the crystal lattice of (I) the molecules of 1 form inversion dimers with head-to-tail π–π-stacking interactions and centroid-centroid distances of 3.579 Å between the thiazolidine and the benzene rings C6 — C11 (Fig. 2). The shortest two intermolecular distances in these π–π-stacks are C3···C7(1 - *x*,1 - *y*,1 - *z*) = 3.2344 (13) Å and C4···C6(1 - *x*,1 - *y*,1 - *z*) = 3.4721 (13) Å. These π–π-stacked pairs are arranged in layers parallel to [001] held together by intermolecular hydrogen bonds O3_phenol_—H3o···O4_carboxyl_(-*x*,2 - *y*,1 - *z*) (Table 1, Fig. 2). To both sides of these layers and separating them at *z*≈ 0, 1, 2, *etc*. are the DMF solvent molecules. They are firmly attached *via* the strong hydrogen bonds O5—H5o···O6, O···O = 2.5529 (10) Å, donated by the COOH group of 1 to the oxygen of DMF. Likely due to this feature the title compound is stable at room temperature against desolvation.

### Experimental

Although the compound 1 (Fig. 3) and its bioactivity was already described (Dayam *et al.*; 2006), a synthesis was not reported. In the present work 1 was synthesized in good yield according to the reaction scheme shown in Fig. 3. A 40% aqueous solution of methylamine (13 g) was added dropwise at 5–7 °C to a solution of 2 (see Fig. 3; 5 g, 28 mmol) in water (110 ml) and stirred for ~2.5 h with gradual warming to room temperature. The aqueous solution was extracted three times with 80 ml portions of EtOAc and freed from residual EtOAc in a rotary evaporator under vacuum at 50 °C. The solution was cooled to 5 °C and acidified with 2 *M* HCl to pH 3. The resulting precipitate was filtered, thoroughly washed with cold water, and dissolved in EtOAc (250 ml). The organic layer was washed with brine, dried (Na_2_SO_4_) and concentrated to give 3.94 g (67%) of 3 (Fig. 3) as colorless solid; m.p. >170 °C (dec.). A solution of compound 3 (3.94 g, 19 mmol) in dry dioxane (60 ml) was refluxed with methyl bromoacetate (2.1 ml, 23 mmol) for 22 h. The solution was concentrated and the resulting yellow solid was dissolved in 0.1 *M* NaOH (100 ml). The solution was washed twice with Et_2_O (100 ml) and acidified with 2*M* HCl. The product was filtered off and dissolved in 1:1 EtOAc-MeOH (300 ml). The organic layer was dried (Na_2_SO_4_) and concentrated to afford compound 4 (Fig. 3) as colorless solid (3.24 g, 68%), m.p. 198–199°C (EtOH). ^1^H NMR (600 MHz, DMSO) δ 7.93 (d, 2H, *J* = 8.3 Hz, H-2, H-6, Ph), 7.03 (d, 2H, *J* = 8.3 Hz, H-3, H-5, Ph), 4.04 (s, 2H, CH_2_), 3.16 (s, 3H, N—CH_3_). ^13^C NMR (150 MHz, DMSO) δ 171.9 (C=O), 167.0 (COOH), 156.6 (SCN), 152.3 (C-1, Ph), 130.8 (C-2, C-6, Ph), 126.6 (C-4, Ph), 121.1 (C-3, C-5, Ph), 32.8 (CH_2_), 29.2 (CH_3_). HRMS (ESI) calcd for C_11_H_10_N_2_O_3_S [M—H]^-^: 249.0339; found 249.0334. A solution of compound 4 (15 g, 60 mmol), 3-ethoxy-4-hydroxybenzaldehyde (12 g, 72 mmol) and sodium acetate (9.8 g, 120 mmol) in glacial acetic acid (300 ml) was refluxed for 4 d. Acetic acid was removed at 140 °C and the suspension was kept at 140 °C over night. After cooling to room temperature, acetic acid was added (100 ml) and the suspension was poured into water (1 *L*) and the resulting precipitate was filtered, washed with water and dried. The residue was crystallized from acetic acid, washed with acetone and dried to give 13 g (54%) of the title compound 1 (Fig. 3) as yellow solid; m.p. >180 °C (dec.). ^1^H NMR (600 MHz, DMSO): δ 9.78 (s, 1H, OH), 7.98 (d, 2H, *J* = 8.5 Hz, H-2, H-6, Ph), 7.70 (s, 1H, C-2, Ar), 7.17 (d, 1H, *J* = 1.9 Hz, =CH), 7.14 (d, 2H, *J* = 8.5 Hz, H-3, H-5, Ph), 6.95 (dd, 1H, *J* = 1.9, *J* = 8.3 Hz, H-6, Ar), 6.89 (d, 1H, *J* = 8.3 Hz, H-5, Ar), 4.04 (q, 2H, CH_2_), 3.33 (s, 3H, N—CH_3_) and 1.31 (t, 3H, CH_3_). HRMS (ESI) calcd for C_20_H_19_N_2_O_5_S [*M*+H]^+^: 399.1009; found 399.1007. Crystals of (I) suitable for X-ray diffraction were obtained by slow evaporation at room temperature of a solution of 1 in dimethylformamide (DMF). Solvents other than DMF, like ethanol, acetone or acetic acid gave only unsuitable material.

### Refinement

C-bonded H atoms were placed in calculated positions and thereafter treated as riding, C—H = 0.95–0.99 Å, *U*_iso_(H) = 1.2–1.5*U*_eq_(C). O-bonded H atoms were refined with AFIX 147 of program *SHELXL97* (Sheldrick, 2008), O—H = 0.84 Å, *U*_iso_(H) = 1.5*U*_eq_(O).

### Crystal data

**Table:**

C_20_H_18_N_2_O_5_S·C_3_H_7_NO	*Z* = 2
*M**_r_* = 471.52	*F*(000) = 496
Triclinic, *P*1	*D*_x_ = 1.386 Mg m^−^^3^
Hall symbol: -P 1	Mo *K*α radiation, λ = 0.71073 Å
*a* = 7.7532 (3) Å	Cell parameters from 9007 reflections
*b* = 9.3081 (3) Å	θ = 2.2–30.0°
*c* = 15.6969 (3) Å	µ = 0.19 mm^−^^1^
α = 86.390 (2)°	*T* = 100 K
β = 89.813 (2)°	Prism, yellow
γ = 87.656 (2)°	0.53 × 0.35 × 0.24 mm
*V* = 1129.61 (6) Å^3^	

### Data collection

**Table:**

Bruker Kappa APEXII CCD diffractometer	6537 independent reflections
Radiation source: fine-focus sealed tube	5993 reflections with *I* > 2σ(*I*)
Graphite monochromator	*R*_int_ = 0.024
φ and ω scans	θ_max_ = 30.0°, θ_min_ = 2.2°
Absorption correction: multi-scan (*SADABS*; Bruker, 2008)	*h* = −10→10
*T*_min_ = 0.89, *T*_max_ = 0.96	*k* = −13→13
29040 measured reflections	*l* = −22→22

### Refinement

**Table:**

Refinement on *F*^2^	Primary atom site location: structure-invariant direct methods
Least-squares matrix: full	Secondary atom site location: difference Fourier map
*R*[*F*^2^ > 2σ(*F*^2^)] = 0.033	Hydrogen site location: inferred from neighbouring sites
*wR*(*F*^2^) = 0.094	H-atom parameters constrained
*S* = 1.02	*w* = 1/[σ^2^(*F*_o_^2^) + (0.0516*P*)^2^ + 0.4019*P*] where *P* = (*F*_o_^2^ + 2*F*_c_^2^)/3
6537 reflections	(Δ/σ)_max_ = 0.002
304 parameters	Δρ_max_ = 0.51 e Å^−^^3^
0 restraints	Δρ_min_ = −0.22 e Å^−^^3^

### Special details

**Table:**

Geometry. All e.s.d.'s (except the e.s.d. in the dihedral angle between two l.s. planes) are estimated using the full covariance matrix. The cell e.s.d.'s are taken into account individually in the estimation of e.s.d.'s in distances, angles and torsion angles; correlations between e.s.d.'s in cell parameters are only used when they are defined by crystal symmetry. An approximate (isotropic) treatment of cell e.s.d.'s is used for estimating e.s.d.'s involving l.s. planes.
Refinement. Refinement of *F*^2^ against ALL reflections. The weighted *R*-factor *wR* and goodness of fit *S* are based on *F*^2^, conventional *R*-factors *R* are based on *F*, with *F* set to zero for negative *F*^2^. The threshold expression of *F*^2^ > σ(*F*^2^) is used only for calculating *R*-factors(gt) *etc*. and is not relevant to the choice of reflections for refinement. *R*-factors based on *F*^2^ are statistically about twice as large as those based on *F*, and *R*- factors based on ALL data will be even larger.

### Fractional atomic coordinates and isotropic or equivalent isotropic displacement parameters (Å^2^)

**Table:**

	*x*	*y*	*z*	*U*_iso_*/*U*_eq_	
S1	0.41930 (3)	0.65538 (2)	0.349946 (14)	0.01698 (6)	
O1	0.80606 (9)	0.38102 (8)	0.35244 (5)	0.02040 (14)	
O2	0.37560 (9)	0.96319 (8)	0.62843 (5)	0.02005 (14)	
O3	0.65049 (10)	0.96754 (8)	0.73138 (5)	0.02233 (15)	
H3o	0.5526	1.0091	0.7342	0.033*	
O4	−0.39179 (10)	0.90766 (9)	0.17578 (5)	0.02725 (17)	
O5	−0.23650 (9)	1.02278 (8)	0.07406 (5)	0.02368 (16)	
H5o	−0.3306	1.0689	0.0647	0.036*	
N1	0.57050 (10)	0.44691 (8)	0.26982 (5)	0.01658 (15)	
N2	0.33188 (11)	0.54753 (9)	0.19732 (5)	0.01936 (16)	
C1	0.43011 (12)	0.54409 (10)	0.26183 (6)	0.01595 (16)	
C2	0.60871 (14)	0.34431 (11)	0.20524 (7)	0.02270 (19)	
H2A	0.7153	0.2885	0.2205	0.034*	
H2B	0.6232	0.3965	0.1496	0.034*	
H2C	0.5133	0.2789	0.2022	0.034*	
C3	0.67482 (12)	0.45592 (10)	0.33963 (6)	0.01610 (16)	
C4	0.60700 (12)	0.56946 (10)	0.39437 (6)	0.01610 (16)	
C5	0.69539 (12)	0.59362 (10)	0.46550 (6)	0.01749 (17)	
H5	0.7951	0.5316	0.4740	0.021*	
C6	0.67052 (12)	0.69420 (10)	0.53172 (6)	0.01676 (17)	
C7	0.52167 (12)	0.78354 (10)	0.54232 (6)	0.01713 (17)	
H7	0.4277	0.7816	0.5037	0.021*	
C8	0.51260 (12)	0.87446 (10)	0.60936 (6)	0.01695 (17)	
C9	0.65368 (12)	0.87976 (10)	0.66581 (6)	0.01783 (17)	
C10	0.79983 (13)	0.79211 (11)	0.65505 (6)	0.01950 (18)	
H10	0.8948	0.7954	0.6929	0.023*	
C11	0.80769 (12)	0.69964 (10)	0.58921 (6)	0.01877 (18)	
H11	0.9078	0.6389	0.5829	0.023*	
C12	0.22371 (12)	0.96318 (10)	0.57563 (6)	0.01843 (17)	
H12A	0.1783	0.8652	0.5764	0.022*	
H12B	0.2514	0.9948	0.5159	0.022*	
C13	0.09223 (13)	1.06669 (11)	0.61232 (7)	0.02253 (19)	
H13A	0.0657	1.0338	0.6713	0.034*	
H13B	−0.0134	1.0705	0.5780	0.034*	
H13C	0.1392	1.1629	0.6115	0.034*	
C14	0.18954 (12)	0.64677 (10)	0.18805 (6)	0.01736 (17)	
C15	0.03664 (13)	0.62483 (11)	0.23377 (6)	0.02102 (19)	
H15	0.0308	0.5477	0.2762	0.025*	
C16	−0.10619 (13)	0.71632 (11)	0.21680 (6)	0.02002 (18)	
H16	−0.2100	0.7017	0.2479	0.024*	
C17	−0.09940 (12)	0.82963 (10)	0.15451 (6)	0.01648 (17)	
C18	0.05412 (12)	0.85138 (10)	0.10947 (6)	0.01830 (17)	
H18	0.0599	0.9289	0.0673	0.022*	
C19	0.19814 (12)	0.76057 (11)	0.12589 (6)	0.01939 (18)	
H19	0.3021	0.7757	0.0950	0.023*	
C20	−0.25683 (12)	0.92308 (11)	0.13672 (6)	0.01868 (18)	
O6	−0.49662 (10)	1.18732 (8)	0.02984 (5)	0.02426 (16)	
N3	−0.76655 (11)	1.27974 (10)	0.05269 (6)	0.02176 (17)	
C21	−0.63956 (13)	1.18080 (11)	0.06532 (6)	0.02044 (18)	
H21	−0.6593	1.1002	0.1038	0.025*	
C22	−0.74498 (16)	1.40627 (12)	−0.00518 (7)	0.0282 (2)	
H22A	−0.7384	1.4918	0.0279	0.042*	
H22B	−0.8436	1.4184	−0.0442	0.042*	
H22C	−0.6384	1.3938	−0.0381	0.042*	
C23	−0.92669 (15)	1.27171 (13)	0.10088 (8)	0.0296 (2)	
H23A	−0.9239	1.1831	0.1381	0.044*	
H23B	−1.0242	1.2713	0.0613	0.044*	
H23C	−0.9399	1.3553	0.1357	0.044*	

### Atomic displacement parameters (Å^2^)

**Table:**

	*U*^11^	*U*^22^	*U*^33^	*U*^12^	*U*^13^	*U*^23^
S1	0.01676 (11)	0.01661 (11)	0.01742 (11)	0.00188 (8)	−0.00169 (8)	−0.00148 (8)
O1	0.0160 (3)	0.0205 (3)	0.0241 (3)	0.0017 (2)	−0.0018 (3)	0.0007 (3)
O2	0.0156 (3)	0.0244 (3)	0.0204 (3)	0.0021 (3)	−0.0045 (2)	−0.0046 (3)
O3	0.0187 (3)	0.0284 (4)	0.0204 (3)	0.0015 (3)	−0.0037 (3)	−0.0071 (3)
O4	0.0177 (3)	0.0369 (4)	0.0265 (4)	0.0031 (3)	0.0047 (3)	−0.0001 (3)
O5	0.0171 (3)	0.0238 (4)	0.0289 (4)	0.0042 (3)	0.0005 (3)	0.0049 (3)
N1	0.0170 (4)	0.0158 (3)	0.0168 (3)	0.0017 (3)	−0.0013 (3)	−0.0016 (3)
N2	0.0195 (4)	0.0195 (4)	0.0187 (4)	0.0026 (3)	−0.0031 (3)	−0.0005 (3)
C1	0.0158 (4)	0.0148 (4)	0.0170 (4)	−0.0002 (3)	0.0000 (3)	0.0007 (3)
C2	0.0264 (5)	0.0214 (4)	0.0203 (4)	0.0046 (4)	−0.0009 (4)	−0.0053 (3)
C3	0.0157 (4)	0.0153 (4)	0.0172 (4)	−0.0025 (3)	−0.0001 (3)	0.0013 (3)
C4	0.0151 (4)	0.0154 (4)	0.0176 (4)	−0.0013 (3)	−0.0001 (3)	0.0006 (3)
C5	0.0160 (4)	0.0176 (4)	0.0187 (4)	−0.0014 (3)	−0.0006 (3)	0.0007 (3)
C6	0.0158 (4)	0.0176 (4)	0.0168 (4)	−0.0021 (3)	−0.0013 (3)	0.0009 (3)
C7	0.0150 (4)	0.0191 (4)	0.0172 (4)	−0.0024 (3)	−0.0030 (3)	0.0011 (3)
C8	0.0147 (4)	0.0184 (4)	0.0175 (4)	−0.0018 (3)	−0.0012 (3)	0.0011 (3)
C9	0.0168 (4)	0.0206 (4)	0.0162 (4)	−0.0030 (3)	−0.0015 (3)	−0.0001 (3)
C10	0.0159 (4)	0.0241 (4)	0.0184 (4)	−0.0011 (3)	−0.0040 (3)	−0.0005 (3)
C11	0.0156 (4)	0.0211 (4)	0.0194 (4)	0.0000 (3)	−0.0025 (3)	0.0003 (3)
C12	0.0155 (4)	0.0204 (4)	0.0194 (4)	−0.0010 (3)	−0.0038 (3)	−0.0007 (3)
C13	0.0184 (4)	0.0230 (5)	0.0262 (5)	0.0011 (3)	−0.0025 (4)	−0.0029 (4)
C14	0.0176 (4)	0.0187 (4)	0.0158 (4)	0.0010 (3)	−0.0037 (3)	−0.0021 (3)
C15	0.0227 (5)	0.0222 (4)	0.0175 (4)	0.0000 (3)	0.0003 (3)	0.0032 (3)
C16	0.0185 (4)	0.0234 (4)	0.0180 (4)	−0.0016 (3)	0.0024 (3)	0.0006 (3)
C17	0.0148 (4)	0.0189 (4)	0.0158 (4)	−0.0003 (3)	−0.0004 (3)	−0.0023 (3)
C18	0.0163 (4)	0.0189 (4)	0.0193 (4)	−0.0002 (3)	0.0006 (3)	0.0019 (3)
C19	0.0157 (4)	0.0210 (4)	0.0210 (4)	0.0001 (3)	0.0012 (3)	0.0017 (3)
C20	0.0163 (4)	0.0214 (4)	0.0186 (4)	0.0002 (3)	−0.0007 (3)	−0.0040 (3)
O6	0.0219 (4)	0.0275 (4)	0.0226 (3)	0.0059 (3)	−0.0010 (3)	0.0004 (3)
N3	0.0206 (4)	0.0220 (4)	0.0226 (4)	0.0031 (3)	−0.0031 (3)	−0.0029 (3)
C21	0.0223 (5)	0.0210 (4)	0.0180 (4)	0.0021 (3)	−0.0045 (3)	−0.0018 (3)
C22	0.0351 (6)	0.0217 (5)	0.0268 (5)	0.0067 (4)	−0.0057 (4)	0.0010 (4)
C23	0.0212 (5)	0.0342 (6)	0.0344 (6)	0.0010 (4)	0.0011 (4)	−0.0103 (5)

### Geometric parameters (Å, º)

**Table:**

S1—C4	1.7564 (10)	C11—H11	0.9500
S1—C1	1.7793 (9)	C12—C13	1.5116 (14)
O1—C3	1.2197 (12)	C12—H12A	0.9900
O2—C8	1.3628 (11)	C12—H12B	0.9900
O2—C12	1.4423 (11)	C13—H13A	0.9800
O3—C9	1.3533 (12)	C13—H13B	0.9800
O3—H3o	0.8400	C13—H13C	0.9800
O4—C20	1.2200 (12)	C14—C19	1.3978 (13)
O5—C20	1.3227 (12)	C14—C15	1.3993 (13)
O5—H5o	0.8400	C15—C16	1.3852 (14)
N1—C3	1.3729 (12)	C15—H15	0.9500
N1—C1	1.3873 (12)	C16—C17	1.3948 (13)
N1—C2	1.4575 (12)	C16—H16	0.9500
N2—C1	1.2671 (12)	C17—C18	1.3978 (13)
N2—C14	1.4118 (12)	C17—C20	1.4863 (13)
C2—H2A	0.9800	C18—C19	1.3874 (13)
C2—H2B	0.9800	C18—H18	0.9500
C2—H2C	0.9800	C19—H19	0.9500
C3—C4	1.4824 (13)	O6—C21	1.2406 (13)
C4—C5	1.3475 (13)	N3—C21	1.3270 (13)
C5—C6	1.4494 (13)	N3—C23	1.4539 (14)
C5—H5	0.9500	N3—C22	1.4563 (14)
C6—C11	1.4015 (13)	C21—H21	0.9500
C6—C7	1.4103 (13)	C22—H22A	0.9800
C7—C8	1.3912 (13)	C22—H22B	0.9800
C7—H7	0.9500	C22—H22C	0.9800
C8—C9	1.4143 (13)	C23—H23A	0.9800
C9—C10	1.3851 (14)	C23—H23B	0.9800
C10—C11	1.3857 (14)	C23—H23C	0.9800
C10—H10	0.9500		
			
C4—S1—C1	91.11 (4)	C13—C12—H12B	110.4
C8—O2—C12	117.96 (7)	H12A—C12—H12B	108.6
C9—O3—H3o	109.5	C12—C13—H13A	109.5
C20—O5—H5o	109.5	C12—C13—H13B	109.5
C3—N1—C1	116.78 (8)	H13A—C13—H13B	109.5
C3—N1—C2	121.81 (8)	C12—C13—H13C	109.5
C1—N1—C2	121.36 (8)	H13A—C13—H13C	109.5
C1—N2—C14	121.40 (8)	H13B—C13—H13C	109.5
N2—C1—N1	120.85 (8)	C19—C14—C15	120.18 (9)
N2—C1—S1	128.47 (7)	C19—C14—N2	118.40 (9)
N1—C1—S1	110.67 (7)	C15—C14—N2	121.13 (9)
N1—C2—H2A	109.5	C16—C15—C14	119.55 (9)
N1—C2—H2B	109.5	C16—C15—H15	120.2
H2A—C2—H2B	109.5	C14—C15—H15	120.2
N1—C2—H2C	109.5	C15—C16—C17	120.75 (9)
H2A—C2—H2C	109.5	C15—C16—H16	119.6
H2B—C2—H2C	109.5	C17—C16—H16	119.6
O1—C3—N1	123.42 (9)	C16—C17—C18	119.36 (9)
O1—C3—C4	125.97 (9)	C16—C17—C20	119.06 (8)
N1—C3—C4	110.60 (8)	C18—C17—C20	121.57 (8)
C5—C4—C3	118.33 (8)	C19—C18—C17	120.46 (9)
C5—C4—S1	130.83 (8)	C19—C18—H18	119.8
C3—C4—S1	110.81 (7)	C17—C18—H18	119.8
C4—C5—C6	133.55 (9)	C18—C19—C14	119.69 (9)
C4—C5—H5	113.2	C18—C19—H19	120.2
C6—C5—H5	113.2	C14—C19—H19	120.2
C11—C6—C7	118.94 (9)	O4—C20—O5	123.61 (9)
C11—C6—C5	115.68 (8)	O4—C20—C17	122.94 (9)
C7—C6—C5	125.38 (8)	O5—C20—C17	113.45 (8)
C8—C7—C6	119.89 (9)	C21—N3—C23	121.33 (9)
C8—C7—H7	120.1	C21—N3—C22	120.97 (9)
C6—C7—H7	120.1	C23—N3—C22	117.53 (9)
O2—C8—C7	126.07 (9)	O6—C21—N3	123.87 (10)
O2—C8—C9	113.74 (8)	O6—C21—H21	118.1
C7—C8—C9	120.18 (9)	N3—C21—H21	118.1
O3—C9—C10	118.26 (9)	N3—C22—H22A	109.5
O3—C9—C8	121.99 (9)	N3—C22—H22B	109.5
C10—C9—C8	119.74 (9)	H22A—C22—H22B	109.5
C9—C10—C11	120.09 (9)	N3—C22—H22C	109.5
C9—C10—H10	120.0	H22A—C22—H22C	109.5
C11—C10—H10	120.0	H22B—C22—H22C	109.5
C10—C11—C6	121.14 (9)	N3—C23—H23A	109.5
C10—C11—H11	119.4	N3—C23—H23B	109.5
C6—C11—H11	119.4	H23A—C23—H23B	109.5
O2—C12—C13	106.71 (8)	N3—C23—H23C	109.5
O2—C12—H12A	110.4	H23A—C23—H23C	109.5
C13—C12—H12A	110.4	H23B—C23—H23C	109.5
O2—C12—H12B	110.4		
			
C14—N2—C1—N1	−179.41 (8)	O2—C8—C9—O3	−0.99 (13)
C14—N2—C1—S1	−1.09 (14)	C7—C8—C9—O3	179.48 (9)
C3—N1—C1—N2	176.75 (9)	O2—C8—C9—C10	178.36 (9)
C2—N1—C1—N2	−0.86 (14)	C7—C8—C9—C10	−1.18 (14)
C3—N1—C1—S1	−1.85 (10)	O3—C9—C10—C11	179.38 (9)
C2—N1—C1—S1	−179.46 (7)	C8—C9—C10—C11	0.01 (14)
C4—S1—C1—N2	−177.56 (9)	C9—C10—C11—C6	0.98 (15)
C4—S1—C1—N1	0.90 (7)	C7—C6—C11—C10	−0.81 (14)
C1—N1—C3—O1	−176.93 (8)	C5—C6—C11—C10	179.63 (9)
C2—N1—C3—O1	0.67 (14)	C8—O2—C12—C13	178.41 (8)
C1—N1—C3—C4	1.95 (11)	C1—N2—C14—C19	108.96 (11)
C2—N1—C3—C4	179.54 (8)	C1—N2—C14—C15	−77.22 (13)
O1—C3—C4—C5	−0.48 (14)	C19—C14—C15—C16	0.23 (15)
N1—C3—C4—C5	−179.32 (8)	N2—C14—C15—C16	−173.49 (9)
O1—C3—C4—S1	177.69 (8)	C14—C15—C16—C17	0.20 (15)
N1—C3—C4—S1	−1.15 (9)	C15—C16—C17—C18	−0.59 (15)
C1—S1—C4—C5	178.01 (10)	C15—C16—C17—C20	178.47 (9)
C1—S1—C4—C3	0.14 (7)	C16—C17—C18—C19	0.56 (14)
C3—C4—C5—C6	178.92 (9)	C20—C17—C18—C19	−178.48 (9)
S1—C4—C5—C6	1.18 (17)	C17—C18—C19—C14	−0.13 (15)
C4—C5—C6—C11	−170.63 (10)	C15—C14—C19—C18	−0.27 (15)
C4—C5—C6—C7	9.84 (17)	N2—C14—C19—C18	173.62 (9)
C11—C6—C7—C8	−0.36 (13)	C16—C17—C20—O4	1.91 (15)
C5—C6—C7—C8	179.15 (9)	C18—C17—C20—O4	−179.04 (10)
C12—O2—C8—C7	0.83 (14)	C16—C17—C20—O5	−177.34 (9)
C12—O2—C8—C9	−178.67 (8)	C18—C17—C20—O5	1.71 (13)
C6—C7—C8—O2	−178.13 (9)	C23—N3—C21—O6	−175.35 (10)
C6—C7—C8—C9	1.34 (14)	C22—N3—C21—O6	−0.22 (16)

### Hydrogen-bond geometry (Å, º)

**Table:**

*D*—H···*A*	*D*—H	H···*A*	*D*···*A*	*D*—H···*A*
O5—H5*o*···O6	0.84	1.73	2.5529 (10)	167
O3—H3*o*···O4^i^	0.84	2.05	2.7386 (11)	139

Symmetry code: (i) −*x*, −*y*+2, −*z*+1.
